# An Exploration of the Unintended Consequences of Performance-Based Financing in 6 Primary Healthcare Facilities in Burkina Faso

**DOI:** 10.34172/ijhpm.2020.83

**Published:** 2020-06-23

**Authors:** Anne-Marie Turcotte-Tremblay, Idriss Ali Gali Gali, Valéry Ridde

**Affiliations:** ^1^École de santé publique de l’Université de Montréal, Montreal, QC, Canada. 2; ^2^Association Action Gouvernance Intégration Renforcement (AGIR), Ouagadougou, Burkina Faso.; ^3^IRD (French Institute for Research on Sustainable Development), CEPED, Université de Paris, Paris, France.

**Keywords:** Performance-Based Financing, Unintended Consequences, Multiple Case Study, Burkina Faso, Diffusion of Innovations Theory

## Abstract

**Background:** Performance-based financing (PBF) is promoted to improve the quality and quantity of healthcare services in low-income countries. Despite the complexity of the intervention, little attention has been given to studying its unintended consequences. Our objective is to increase evidence on the unintended consequences of PBF in Burkina Faso.

**Methods:** Using the diffusion of innovations theory, we conducted a multiple case study. The cases were 6 healthcare facilities in two districts. Between April 2015 and 2016, we collected data through 101 semi-structured interviews, discussions, observations, and documents. We conducted thematic analysis using a hybrid deductive-inductive approach. Secondary data was used to illustrate the evolution of reported services. We conducted a cross-case synthesis to identify the results arising independently from more than 1 case.

**Results:** A desirable unintended consequence of PBF was that 3 facilities limited the sale of non-prescribed medication to encourage patients to consult. Undesirable unintended consequences were found in the majority of facilities including fixation on measures rather than on underlying objectives, the pursuit of narrow and less relevant performance indicators, gaming, and teaching trainees improper practices. Providers in all facilities deliberately manipulated medical registers and documents, such that the reported quantity and quality of care differed from what was actually delivered. While most participants indicated that PBF was more advantageous than previous practices, the long payment delays were a source of dissatisfaction and demotivation across all facilities. Dissatisfaction also emerged in relation to the distribution of subsidies and the non-attribution of quality points for services delivered by certain staff considered "unqualified" in guidelines. Results in many facilities revealed suboptimal planning, a perception of the intervention as "budgetivorous," as well as tensions related to the principle of managerial autonomy.

**Conclusion:** PBF led to numerous unintended consequences that could undermine the intervention’s effectiveness. The findings contribute to a more comprehensive picture of the consequences of implementing PBF. Policy-makers can use the results of this study to devise effective strategies before, during and after the implementation of the intervention to minimize undesirable unintended consequences and promote desirable ones.

## Background

**Key Messages**

**Implications for policy makers**

- Policy-makers and donors should carefully consider the breadth and scope of unintended consequences before pursuing or scaling up performance-based financing (PBF) interventions.
- Reducing undesirable consequences of PBF may require some adjustments to transfer subsidies on time, ensure that their distribution is perceived as equitable amongst actors involved, improve the staff members’ internalization of quality standards, adapt performance indicators to the local context or seek truly independent PBF auditors.
- Policy-makers should be wary of incentives and performance pressure that can encourage the deliberate and systematic falsification of medical registers.
- Program planners should increasingly monitor desirable and undesirable unintended consequences of PBF to gain a more comprehensive understanding of its impact on health systems.

**Implications for the public**

There is an urgent need to improve healthcare systems in low-income countries. Governments and donors are increasingly implementing performance-based financing (PBF) to improve the quantity and quality of care. Healthcare facilities receive a unit fee for each service provided and bonuses based on the quality of care. We found that PBF led to numerous unintended consequences such as gaming and fixation on performance indicators rather than on underlying objectives. Providers spend considerable amounts of time falsifying registers to improve their performance scores. Dissatisfaction grew amongst staff due to the lateness of bonuses and the distribution modalities of premiums. Many people viewed the intervention as too costly. These findings will be useful to develop strategies that help prevent or minimize unintended consequences in order to successfully improve the healthcare systems’ performance.

Improving healthcare systems performance is key to achieving universal health coverage by 2030. The World Health Organization (WHO) is encouraging low- and middle-income countries (LMICs) to move from passive to strategic purchasing of health services.^1^ Performance-based financing (PBF) is one means of introducing elements of strategic purchasing.^2^ With PBF, facilities can receive a unit fee for each targeted service provided, as well as bonuses conditional on quality of care.

While PBF is rapidly expanding in LMICs, many public health actors hypothesize it can have important unintended consequences that influence its overall effectiveness. These unintended consequences are defined as “changes for which there is a lack of purposeful action or causation that occur to a social system as a result of an innovation.”^3^ These changes can be desirable or undesirable, as well as anticipated or unanticipated, depending on the stakeholders’ perspectives.

Although it often sparks debates, research on the unintended consequences of PBF remains scarce. In high-income countries, a synthesis of reviews found some evidence that PBF was associated with risk selection, spillover effects, gaming behavior, and changes in the providers’ intrinsic motivation.^4^ Yet, after examining 12 pay for performance programs in high-income countries, Cashin et al^5^ concluded that unintended consequences were never carefully assessed.

In LMICs, at least 2 literature reviews have demonstrated the lack of evidence documenting the unintended consequences of PBF.^6,7^ However, some empirical evidence is beginning to emerge. Basinga et al^8^ suggested PBF in Rwanda had the greatest impact on services requiring less effort. Other studies in the country found that information was regularly distorted, that providers used gaming strategies and that consultations were rushed to reach targets.^9,10^ A study in Cameroon found that PBF raised concerns regarding drug quality and inequities between facilities.^11^ In Uganda, Benin, and Burkina Faso, studies highlighted that audits generated overwhelming workloads.^12-14^ A study on PBF community verifications revealed falsification of data, loss of patient confidentiality, and fears among patients.^14^ In Malawi, PBF had both positive and negative effects on the health workers’ basic psychological needs for autonomy, competence, and relatedness, which are central to intrinsic motivation.^15^ In Ghana, Aninanya and colleagues’^16^ qualitative findings indicated that performance-based initiatives enhanced the providers’ reported pride, although the quantitative results on this were not significant. More recently, Macarayan and colleagues’^17^ study in Ghana found that women who sought care in facilities with management scores at the 90th percentile rated their waiting times as worst but reported higher levels of trust compared to women attending facilities in the 10th percentile. In the Democratic Republic of Congo, Maini et al^18^ showed that the removal of PBF negatively affected many dimensions of motivation for staff members. However, an important gap in the literature remains because none of these studies specifically aimed to explore all the potential unintended consequences that could emerge. Thus, they did not use any frameworks and methods that enabled them to fully capture these phenomena.

There are several reasons for studying the unintended consequences of PBF in LMICs. The likelihood that PBF triggers unintended consequences going well beyond the objectives of the intervention is high. There is a lot of uncertainty about how new practices will function in complex systems such as healthcare systems.^19^ Although they may be less discernible, unintended consequences may be far-reaching and as equally important as intended consequences. Stakeholders must have a comprehensive understanding of both the intended and unintended consequences in order to judge the value of an intervention.

 This paper is intended to fill a knowledge gap on the neglected topic of unintended consequences of PBF in LMICs. A pilot PBF test implemented in Burkina Faso to improve the healthcare system’s performance provided a unique opportunity to develop evidence on the unintended consequences of PBF in a real-life setting. We posed the following research question: What are the unintended consequences of PBF, and their contributing factors, in primary healthcare facilities in Burkina Faso?

###  Theoretical Framework

We based our theoretical framework on Rogers’ diffusion of innovations theory^19^ for several reasons. While it has proven its utility to analyze the consequences of health innovations^3,20^ it constitutes an original approach to study PBF in LMICs. It is also one of the rare theories that provides a detailed typology of consequences (see below) while remaining sufficiently flexible to be applicable to any innovation. Moreover, it is comprehensive by taking into account the entire diffusion process of innovations as they course through the structure of a social system.

The theory postulates that the implementation of innovations such as PBF does not always conform to plan.^19^ Adopters (eg, healthcare providers) often modify the innovation to suit the organization’s needs and structure, just as the organization’s structures are altered to fit the innovation. Change agencies (eg, ministries of health), which promote innovations when they perceive a performance gap, can offer financial incentives to hasten their adoption. According to the theory, the main purpose of incentives is to give the new practice a relative advantage over previous ones. Although incentives increase the quantity of innovation adopters, the quality of adoption may be low, thus limiting the intended consequences. Key variables influencing the diffusion process pertain to: (1) the nature of the social system; (2) its members’ characteristics; (3) the nature of the innovation; and (4) the use of the innovation.

**Figure 1:**
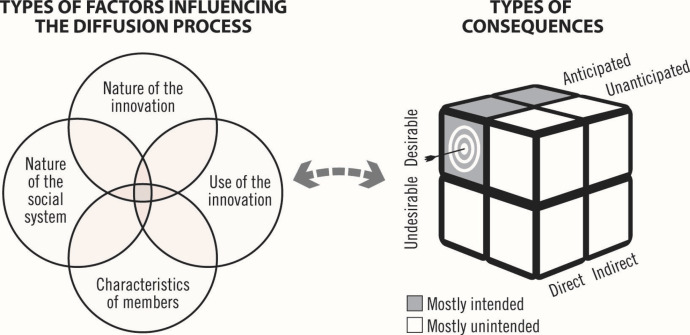
Conceptual Framework. Adapted from Rogers,^19^ Bloomrosen et al,^20^ and Turcotte-Tremblay et al.^14,26^

Inspired by Rogers,^19^ we classified consequences into 3 categories: (1) desirable or undesirable; (2) direct or indirect, depending on whether the changes related to processes or outcomes; and (3) anticipated or unanticipated. We considered a consequence to be anticipated if it was addressed in the implementation guides or if it was in line with the “spirit of the intervention” or its “ideas” (ie, beliefs, assumptions or perceptions),^21^ according to PBF experts. We further refined Rogers’ classification by considering that the following types of consequences tend to be unintended by program planners: undesirable/anticipated, undesirable/unanticipated and desirable/unanticipated. Our rationale for classifying these consequences as unintended was that program planners are not likely to purposefully target changes they consider undesirable or have not anticipated. Like Bloomrosen et al,^20^ we expected that consequences that are desirable/anticipated would tend to be intended by program planners. Similar to what Jabeen^22^ previously argued, program planners trying to promote a new intervention are likely to have listed and exhausted all the desirable outcomes. We did consider that some desirable/anticipated consequences could be unintended if they were, for example, positive spillover effects that were foreseen but not initially targeted by program planners. This conceptualization is consistent with recent literature suggesting that unintended consequences can be either anticipated or unanticipated as well as desirable or undesirable.^23-25^ Figure 1 illustrates our framework. Its applicability has been presented elsewhere.^14,26^

## Methods

###  Study Setting

The study took place in 2 rural districts of Burkina Faso where improving the healthcare system’s performance remains a challenge. The low quality of healthcare is often characterised by the staff’s inhospitality, insufficient equipment/medication and lack of training.^27^

In 2011, the government of Burkina Faso, with World Bank support, conducted a pre-pilot PBF test in 3 districts to address generalized quality deficiencies and improve healthcare system performance.^28^ According to Steenland et al,^29^ this intervention changed the previous financing system by defining a package of key health services to be targeted at contracted facilities, and issuing payments based on quantity and quality for these services. However, this pre-pilot PBF test did not include some recommended elements of PBF including an increase in health facility autonomy and the introduction of improved management tools.^29^ Thus, in 2014, the intervention was modified to incorporate these elements and expanded to 12 more districts in order to conduct an experimental impact evaluation funded by the World Bank. The specific objectives of the intervention were to (1) increase the utilisation of healthcare services; (2) improve the quality of healthcare services; (3) improve the efficiency of the healthcare system; (4) ensure equity in access to healthcare services; (5) reinforce the motivation of personnel; (6) improve community participation; (7) reinforce the health information system; (8) consolidate public – private partnership; and (9) reinforce the governance of the healthcare system. Four intervention arms were implemented combining PBF modalities with different unit fees for service and equity measures (Supplementary file 1). The intervention model is available online.^30^

Each month, a PBF auditor from the contractualisation and verification agency counted the number of reported healthcare services in registers to establish the subsidies. In total, 23 indicators were subsidized for the quantity of care at the primary care level. Every trimester, a team composed of district management team members (eg, doctor, midwife, nurse and pharmacist) assessed the facilities’ technical quality of care by sampling records from various medical registers and observing the facilities’ environment. Quality scores were reported in a 113-item grid (covering 28 domains) and were used to determine the bonuses to be paid (Supplementary file 2). Community verifications were also supposed to be conducted every trimester. Subsidies and bonuses were used to pay for facility expenditures and premiums to motivate staff. Providers were required to use an index tool every month to update the facilities’ revenues, plan expenditures, and determine the distribution of premiums. Each trimester, providers were also required to produce a performance improvement plan to set objectives and plan activities.

###  Study Design

We conducted a multiple case study with several embedded levels of analyses, using both qualitative and quantitative data.^31^ Case studies are useful to investigate contemporary phenomenon in depth and within their real-life context when the boundaries between the phenomenon and context are not clear.^31^ Evidence from multiple cases is considered more robust because it enables replication.^31^ For this study, the cases were 6 primary healthcare facilities, called *Centres de santé et de promotion sociale* (CSPS) located in 2 districts. We selected 2 of the 12 districts involved in PBF in Burkina Faso. We selected these 2 districts because they (1) were located in diverse regions, (2) represented the normal context of the healthcare system, and (3) were relatively safe for data collectors. The first district has 21 primary healthcare facilities and 1 medical center with surgical satellite services. The second district has 56 primary healthcare facilities and 1 medical center with surgical satellite services and 1 regional hospital. Case selection for healthcare facilities followed a multistage screening procedure described elsewhere.^32,33^ Table 1 describes each primary healthcare facility included.

**Table 1:** Description of 6 Cases Included

**Descriptors**	**Pilot Cases**		**Primary Cases**			
	**Facility A**	**Facility B**	**Facility 1**	**Facility 2**	**Facility 3**	**Facility 4**
Intervention arm	PBF 2	PBF 2	PBF 3	PBF 3	PBF 1	PBF 1
Initial performance	Low	High	Low	High	Low	High
PBF payments owed or transferred between trimester 1 2014 and trimester 1 2016^a^	10 117 781 F CFA	12 909 022 F CFA	9 389 071 F CFA	6 450 040 F CFA	12 610 680 F CFA	6 412 805 F CFA
Average scores for quality verifications between trimester 1 2014 and trimester 1 2016^a^	74%	84%	71%	70%	86%	67%
Staff members	2 nurses, 1 auxiliary midwife, 1 drug manager, 2 janitors, 1 guard, 2 trainees (temporary)	1 nurse, 2 auxiliary midwives, 1 IHW, 1 drug manager, 2 janitors, 1 guard, 5 trainees	1 nurse, 1 auxiliary midwife, 2 IHWs, 1 drug manager, 1 janitor, 1 guard, 1 IHW volunteer	1 nurse, 1 auxiliary midwife, 1 IHW, 1 drug manager, 1 janitor, 1 guard	2 nurses, 1 midwife, 2 IHWs, 1 auxiliary midwife, 1 drug manager, 1 guard, 1 janitor, 3 trainees	1 nurse, 1 auxiliary midwife, 1 IHW, 4 trainees
No. of villages	8	10	5	8	22	6
Population in catchment area	8900 people	7700 people	8000 people	3600 people	11 000 people	3700 people

Abbreviations: PBF, performance-based financing; IHW, itinerary health worker. ^a^ Data available online: http://www.fbrburkina.org/data.

###  Sampling for Interviews 

Participants included a wide range of stakeholders, including providers (ie, nurses, midwives, itinerary health worker), support staff (ie, drug manager, janitors, guards), patients (eg, seeking care for curative consultations or maternal and child health), and community representatives (eg, members of the facility management committee) (see Table 2). Participants were purposefully selected based on their ability to provide relevant information and their accessibility. In each facility, we selected all the providers, support staff and volunteers for semi-structured interviews. Then, following the snowball approach, some participants referred us to other people who could shed light on the intervention.^34^ This strategy was used to identify potential participants who were knowledgeable about or had a particular experience with the intervention (eg, auditors and administrative staff at the district level). Overall, we conducted 101 semi-structured interviews.

**Table 2:** Demographic Characteristics of Interview Participants

**Characteristics**	**n **
Gender	
Females	31
Males	70
Place of residence	
Rural	84
Semi-urban	15
Urban	2
Education	
Primary or less	29
Secondary	29
Post-secondary	43
Age group	
18-30	16
31-50	80
51-70	5
Status	
Facility level	
Providers	21
Support staff (drug manager, janitor, guard)	15
Volunteers and trainees	7
Community leaders (eg, COGES, CHWs, counselors)	25
Patients	16
District level	
Administration (eg, manager, accountant)	4
Contracting and verification agents (auditors)	4
Members of associations conducting community verifications	7
National level	
Representative from the PADS	1
Representative from the results-based financing – technical service	1

Abbreviations: COGES, facilities’ management committees; CHWs, community health workers; PADS, program to support health development.

###  Data Collection 

 We adopted a broad, exploratory approach in order to capture all changes that were not initially targeted by program planners. Through observation, semi-structured interviews and informal discussions, we collected data on various dimensions of the healthcare system including service delivery, governance, human resources, medication, health information system and financial management.

 Data were collected during 3 sequential phases, with each informing methods for the next. In the pilot phase (April 2015), the first author conducted one week of fieldwork in 2 facilities in the same district (facilities A and B). These served as pilot case studies to validate the feasibility of the methods. In phase 1 (January–April 2016), the first author conducted 3 months of fieldwork, examining 4 facilities in another district with longer field visits and more participants, for greater depth (facilities 1–4). Each facility was visited for 2 weeks. The first week primarily served to conduct observation within the facilities and the second week served to conduct semi-structured interviews with participants. The first author lived in the facilities which enabled her to conduct observation as well as informal discussions around the clock. The first author also attended a 6-day annual national PBF review meeting for 2015. In phase 2 (May 2016), the second author conducted 20 days of fieldwork in those facilities and neighbouring ones, to deepen the assessment of community verifications.

Of the 101 semi-structured interviews conducted, 11 were in the pilot phase; 76 in phase 1; and 14 in phase 2. Our semi-structured interview guides^35^ built upon previous questionnaires used for research on diffusion of innovations^36,37^ but were tailored to our objectives and participants (see Supplementary file 3). They enabled us to assess how factors related to the social system, characteristics of the members, and the nature and use of the intervention interacted to produce consequences over time. Interviews were recorded and local research assistants made verbatim transcriptions.

 In total, 258 observation sessions were recorded in research diaries. Observations sites included health facilities and villages. During observation, we collected a wide range of intervention documents (eg, quantity and quality verification reports, index tools) to fuel our analyses.

We also used publicly available secondary quantitative data on service delivery (http://www.fbrburkina.org). These longitudinal data were collected monthly in each facility for PBF audits. Healthcare workers reported the quantity of healthcare services that were recorded in the medical registers. Then, PBF auditors verified the reported data by manually recounting the quantity of services. They entered the data into an electronic platform. We accessed and used data collected between 2014 and 2016 on the number of integrated household visits (IHVs) and the number of people who underwent voluntary HIV screening.

We used several strategies to increase the trustworthiness of findings including (1) prolonged engagement on the field, (2) peer debriefing with members of the research team, (3) collection of audio recordings and photographs to test findings, (4) triangulation between sources of information and methods, and (5) member checks with stakeholders to confirm results.^38^

###  Data Analyses 

The primary unit of analysis was each healthcare facility. We combined deductive and inductive thematic analysis.^39^ We began by developing a template of themes based on our theoretical framework. Then, we carefully read the transcripts of the recorded semi-structured interviews and field notes to assign the raw data to the predefined themes. At the same time, we derived new themes that emerged from the data and were judged relevant to our research topic. Data were triangulated by comparing different information sources.^40^ QDA Miner, a qualitative data analysis software, was used to code and retrieve text segments. We integrated the results from all data collection phases and used a cross-case synthesis to draw general conclusions. Following a replication logic for multiple case studies, we considered results arising independently from more than one case to be more powerful than those from a single case, and gave the former more importance in the results.^31^ To avoid cherry-picking results within the rich material, we only present unintended consequences that emerged in multiple healthcare facilities. We organized a member check in Burkina Faso to confirm the researchers’ data interpretation, triangulate results, and validate conclusions.^41^ Further member checks were conducted subsequently on specific elements. We selected verbatim quotations from participants and field notes to enable readers to access the raw data and assess the credibility of results.^38^

## Results

Table 2 presents descriptive statistics of participants. We conducted semi-structured interviews with 101 participants: Thirty-one were women and 70 were men. This discrepancy is due to gender inequalities in the workplace. Over a third of interviewees (n = 36) worked in facilities either as providers or support staff. Almost a quarter of interviewees (n = 25) were community leaders involved in the healthcare system. At the facility level, we interviewed 21 providers, 15 support staff, 7 trainees or volunteers, 25 community leaders and 16 patients. Almost 80% of participants interviewed were between 31 and 50 years of age because we predominantly interviewed people in the healthcare system’s workforce and we did not attempt to conduct interviews with minors.

PBF led to important unintended consequences, classified according to our model in Table 3. In the subsections below, we explain how the nature and use of the intervention interacted with the actors’ characteristics and the nature of the social system to cause these unintended consequences.

**Table 3:** Classification of Unintended Consequences

	**Anticipated**		**Unanticipated**	
	**Direct (Process)**	**Indirect (Outcome)**	**Direct(Process)**	**Indirect (Outcome)**
Desirable	♦ No unintended consequence detected in this category	♦ No unintended consequence detected in this category	• Limits on medication sales without consultations	♦ No unintended consequence detected in this category
Undesirable	• Gaming • Fixation on indicators and subsidies • Falsification of medical registers and documents • Complacency, collusion and complicity	♦ No unintended consequence detected in this category	• Teaching trainees improper practices • Overwhelming paperwork • Pursuit of narrow performance indicators • Manipulation of index tools • Tensions and conflicts related to index tools • Staff’s dissatisfaction and demotivation due to payment delays • Suboptimal planning due to payment delays • Financial issues • Frustrations for providers not eligible for quality points • Tensions between managerial autonomy and top-down control • Activities delayed and reduced due to gradual withdrawal of other funding • A “budgetivorous” intervention	♦ No unintended consequence detected in this category

Supplementary file 4 specifies how the anticipated consequences were addressed in the intervention guides.^30,42^ Note that intended consequences are not included in this analysis. According to our framework, the dark and light grey segments indicate “mostly intended” and “mostly unintended” consequences, respectively.

###  Desirable and Unanticipated

####  Limits on Medication Sales Without Consultations

 Three facilities adopted medication-related strategies to increase the number of consultations recorded in registers. In 2 facilities, staff refused to sell medications to people who did not first consult a provider. In another, the head nurse doubled the cost of medication for people who did not consult providers. Locally, these were perceived as desirable practices that would reduce self-medication. However, a drug manager reported that a small number of patients left without consulting because they could not afford additional costs.

“*Before PBF, many people came to buy drugs but few went for consultations so the head nurse requested that everyone gets a consultation. That way, we can record them in the registers which increases the quantity score when the PBF verifier comes*” (Facility 3, drug manager, observation notes).

###  Undesirable and Anticipated

####  Gaming

 Providers and staff members in most facilities adopted gaming strategies, defined as deliberate manipulation of behaviour to secure strategic advantage. One common strategy involved staging facilities when PBF audits were announced. Medication managers in 2 facilities reported keeping medication boxes on the floor and placing them on shelves just before PBF auditors arrived to get quality points. Medication stock cards were updated before verifications. Janitors reported working more when informed that auditors were coming. Another example of staging were the extra lab coats with identity badges that providers made to meet PBF criteria. Although providers often received high scores for their attire, our observations in all the facilities showed they usually did not wear these coats in their daily practice due to heat. These gaming strategies, adopted in multiple facilities, were instrumental in obtaining PBF points and bonuses.

“*My coat is heavy when it’s hot. The days that they [PBF auditors] come, though, I wear it so as not to lose points” *(Facility 1, drug manager, interview 22).

####  Fixation on Indicators and Subsidies

 PBF sometimes encouraged a narrow emphasis on indicators rather than underlying objectives. For example, some facilities installed curtains to meet PBF confidentiality criteria. Providers often received excellent scores for visual privacy. Yet our observations showed patient confidentiality was regularly compromised, with multiple patients examined simultaneously in most facilities. Moreover, untrained individuals (eg, guards, friends) freely entered consultation rooms, breaching confidentiality.

 Staff members appeared fixated more on paperwork than on care provision. In facility 3, for example, a nurse falsified the register for integrated management of childhood illnesses (IMCIs) while unqualified staff treated a child. In facility 4, the medication manager falsified records before a PBF audit while providers sold medication directly to patients (a prohibited practice).

 Another example of fixation was that providers in all the facilities regularly filled out many medical registers and new PBF management documents retrospectively (sometimes weeks later) with arbitrary or approximate information to satisfy PBF criteria. In all facilities, growth curves were systematically filled out retrospectively, limiting their utility in clinical practice. Incomplete registers automatically received a score of zero during PBF audits, so providers often invented information to fill in blanks. While some retrospective filling of registers had occurred before PBF, it was now done more systematically to avoid leaving any blanks. Some providers openly admitted doing more retrospective filling with PBF so they would not be shamed or outperformed by other facilities in the reporting of scores.

“*The manager, at the end of each month, tells me he has to update his papers to be compliant so that the other CSPSs don’t outdo him…. he explains to me that they received such-and-such a resource”* (Facility 3, management committee member, interview 79).

 Providers also displayed fixation on subsidies. For example, providers in all facilities were fixated on the number of paid IHVs, perceived as a “quota” not to be exceeded. In facility 3, the head nurse expressed discontent when IHVs were disqualified during a PBF audit because he “lost money,” but displayed no concern regarding their poor quality, the reason for disqualification. Fixation on subsidies also motivated providers’ threats to stop certain activities if PBF payment delays continued.

“*Before PBF, people just worked, no one complained; now, with PBF, all people talk about is subsidies, subsidies, and it’s become a kind of obsession that’s a constant hassle” *(National manager, interview 105).

####  Falsification of Medical Registers and Documents

Providers across facilities deliberately manipulated medical registers and documents, such that the reported quantity and quality of care differed from what was actually delivered (Supplementary file 5). Providers in all facilities routinely modified documents ahead of audits to meet PBF criteria. This falsification was time-consuming and conducted openly. We were able to infer causal relationships between PBF and falsification of registers by combining complementary evidence: (1) providers explicitly referred to PBF while falsifying registers; (2) some routinely falsified registers were created specifically for PBF; (3) some of the falsification was conducted in preparation for PBF quantity and quality audits; and (4) PBF audit reports showed providers were initially criticized for not filling out registers, which were then falsified during later stages. Ultimately, falsification of registers and documentation was instrumentalized to obtain higher scores and subsidies, as highlighted by this midwife who was repeatedly observed falsifying partographs and other medical registers during the course of this study:

“*The first time that the PBF auditors came, I had 30 partographs and I was given a score of zero everywhere. I almost shed tears. It hurt me a lot because I had taken the blood pressure every 2 hours and they said that it should be every 4 hours (…) Since that day, I always get a score of 100%” *(Facility 4, provider, interview 85).

 Participants reported numerous factors that explained this practice, such as pressure to perform, competition between facilities, implementation challenges (eg, shortage of qualified staff, time required to complete registers), strict PBF criteria unadapted to local realities (eg, lengthy forms with no leeway for omissions), and desire for premiums. Moreover, some registers had not been part of the providers’ daily practice before PBF. Providers sometimes dismissed the registers as “papers” (ie, externally imposed bureaucracy) and explained that they did not, in fact, subscribe to their importance. They also reported that some registers did not serve their needs.

 Audits did not always detect falsification. Providers entered false consultations directly into medical registers, then manually counted the numbers of monthly services (real and false). They declared these numbers during audits. PBF auditors checked these numbers by manually recounting the services reported in the same medical registers. Because the original source of information (ie, registers) had been tampered with, auditors often could not distinguish between real and false consultations. Occasional differences detected between numbers declared by providers and auditors’ validated numbers usually reflected calculation errors related to manual counting rather than falsification attempts (which remained undetected).

 Some participants at the district and national levels reported being aware of the falsification of registers. PBF auditors were trained to look for signs that data had been falsified, such as use of the same pen or corrector fluid. Providers adapted their falsification strategies accordingly to avoid detection. Auditors explained they were unable to determine whether patients truly received services reported in the registers because they did not observe care in real time.

Table 4 presents the types of services routinely falsified, including registers for IMCIs, IHVs, and maternity ward consultations. Below, we present examples of the various types of falsifications for incentivized services.

**Table 4:** xamples of Falsified Healthcare Services or Information to Qualify for PBF Subsidies or Bonuses

**Services Falsified**	**Examples of Citations**
IMCI	*“On seeing the drug manager filling out the IMCI register at his home, with no patients, a midwife from a neighbouring facility asked, “You are filling those out because of PBF, [aren't you]?” The drug manager mumbled a response. The midwife quickly said, “I’m not a PBF auditor!” and changed the subject”* (Facility 4, observation notes). *“Providers systematically enter children in the register and consider those children to have been managed with the IMCI approach, even if the IMCI procedure was not used. Some districts even have 100% of consultations using the IMCI approach, which is false … there’s money to be made with PBF, so there are risks of fraud” *(National manager, discussion).
Partographs	*“Yes, I do deliveries and when I do, I don’t use the partograph. I put the time of arrival, I do the delivery, and when the birth attendant comes, she does her partograph.… Because if I do the partograph, the PBF auditors will invalidate it [give a score of zero] because I’m an itinerant health worker and I’m not supposed to do deliveries”* (Facility 4, provider, interview 9). *“On Sunday, March 13 … the birth attendant sat on her mat with the partograph register. She filled out partographs for March 8 and March 11 from A to Z …. For the delivery on March 11, the birth attendant was not working at the facility. She was at a wedding in another city”* (Facility 2, observation notes).
Integrated household visits	*“The drug manager and the IHW trainee sat down to finalize the household visit forms because the PBF auditor was supposed to be there at 3 pm for the quantity audit. They were stressed! ‘Give me a date!’ the manager said to the IHW trainee. He randomly added about a dozen dates for visits and another dozen for follow-up appointments. Then he signed for the community health workers and even for heads of households. The other trainee arrived and asked, ‘What, lying again!?’… The drug manager counted the forms and realized that the strong and weak points and the analysis had not been filled out.… He asked me to fill out the forms, even though I wasn’t present during the visit and am not trained as a health provider” *(Facility 4, observation notes).
Consultations in maternity ward	*“Three providers met at the itinerant health worker’s home to count the number of consultations for the PBF audit that was to take place in 2 days. They started at 6:13 pm and ended at 10:16 pm … ‘It’s low, low,’ the head nurse said to the birth attendant on seeing the number of children between 12-23 months seen in consultation… The itinerant health worker said, ‘We just have to add in the register for those who didn’t come. We’ll fix it.’ The head nurse replied, ‘We’ll count first and fix afterward, if need be.’ At one point, the head nurse added a consultation. He filled in an entire column, even though we had no patients”* (Facility 2, observation notes). *“The midwife filled out the postnatal consultation register using the birth register. She filled in several consultations with no patients there … She left the maternity ward saying, ‘PBF gets on my nerves! Just hearing the name gives me a headache!’” *(Facility 3, observation notes). *“The birth attendant was filling out the prenatal consultations register in the maternity ward. She added at least 10 consultations, even though there were no patients or pregnant women near her”* (Facility 3, observation notes).
Appointment dates	*“Some women are illiterate. We try to tell them the appointment is in 4 Thursdays, but sometimes they come a few days early. Some villages are more than 10 km away round-trip. Those women come by bicycle or even on foot if their husbands aren’t there. We can’t tell those women it’s the wrong day, come back in 5 days, because they won’t come back. But if we enter the real date, we’ll be penalized by PBF because it’s not one month later. So we don’t write that date … There are reasons for low attendance that aren’t due to the providers’ motivation, such as illiterate women or the distances of villages. PBF should have more flexible criteria for that”* (Facility 3, provider, observation notes).
Providers’ identity and qualification	*“The head nurse recopied all the consultations into the real register and signed as if he had delivered the services. But he was in another city…. He made corrections as he went along*” (Facility 2, observation notes).
HIV screening	*“There were also overdeclarations...”* (National manager, discussion). “*We can’t even figure out where the reagents the providers use come from. They’re not from the healthcare system” *(National manager 106, discussion).
Prescriptions	*“What we’ve seen is that sometimes [providers] report in registers having prescribed what the [diagnostic and treatment] Guide recommends, but really they’ve prescribed something else*” (National manager 106, discussion).
Other health data	*“The midwife came and added at least 3 prenatal consultations, even though there were no pregnant women here. One of the additions was for March 25, but it was March 26. Then, she counted the total number of prenatal consultations for their monthly report to be submitted to the district management team”* (Facility 3, observation notes).
Absences	*“The book is there, but absences are not noted”* (Facility 1, staff, interview 23).

Abbreviations: PBF, performance-based financing; IMCI, integrated management of childhood illness; IHW, itinerary health worker.

**Figure 2:**
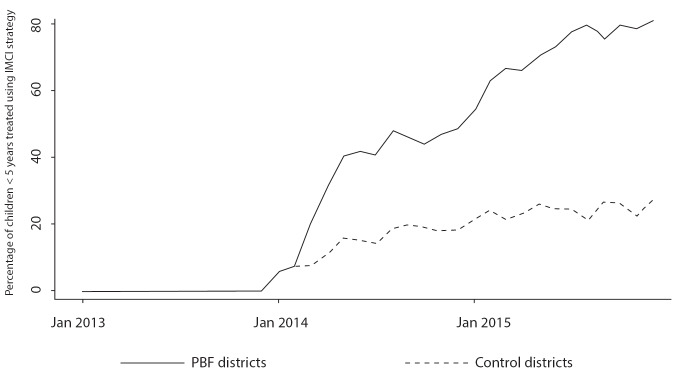
Percentage of Children Treated Using the IMCI Approach in PBF and Control Districts.^43^ Abbreviations: PBF, performance-based financing; IMCI, integrated management of childhood illness.

IMCI: In an annual PBF meeting, Figure 2 was presented to show that, in facilities in PBF districts, the percentage of children treated using the IMCI strategy increased after PBF was implemented (January 2014) compared to facilities in other districts. Participants at the local and national levels argued that the increase in the utilization of the IMCI strategy was one of the main strengths of PBF. Our observation, however, showed that IMCI registers, which determined about 10% of PBF quality scores, were consistently falsified and filled out retrospectively in at least 3 facilities; they were never used or filled out during consultations, despite the fact that some questions required patients’ input. Those registers were often filled out by another provider than the one who provided care and subsequently signed by a provider who met the qualifications for PBF audits. In facility 4, for example, the drug manager filled out the IMCI registers at his house during his free time, even though he was neither qualified nor present during consultations.

**Figure 3:**
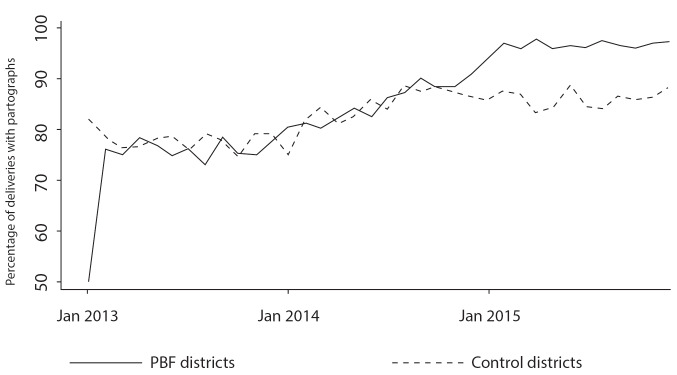
Percentage of Births Conducted Using a Partograph in PBF Districts Compared to Control Districts.^43^ Abbreviation: PBF, performance-based financing.

Partograph: According to the official discourse of local actors, the percentage of births conducted using a partograph increased considerably due to PBF (see Figure 3 presented in an annual PBF meeting). Facilities often received high quality scores for the “proportion of births followed with the help of a partograph” and for “quality of the surveillance for labour and delivery.” Observation in the majority of facilities, however, showed that partograph registers were not routinely used during the childbirth process, despite the fact that some information needed to be reported in a timely fashion to guide clinical decisions. Data reported in partographs were estimated or invented to meet quality evaluation criteria. Partographs were commonly filled out by a qualified provider who did not necessarily attend the birth, sometimes days after the delivery, just before PBF audits. In case 2, for example, the birth attendant calmly filled out multiple partographs in her home, while sitting on a mat, drinking tea. She created a false partograph for a delivery conducted by the itinerant health worker, in her absence, to get PBF quality points. In case 4, the itinerant health worker explicitly explained how the partographs were falsified for PBF verifications.

**Figure 4:**
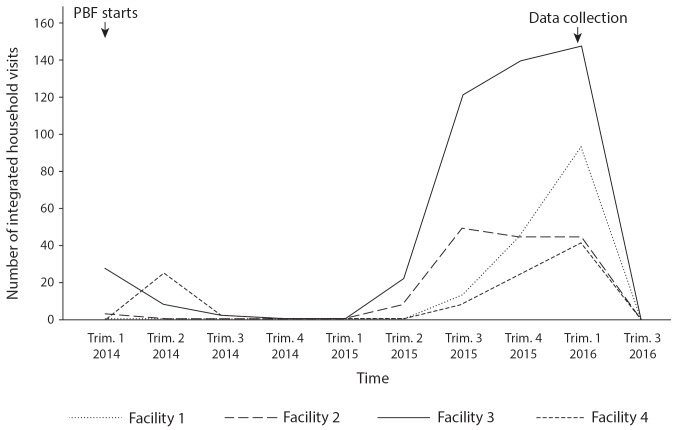
Number of IHVs Per Trimester for 4 Main Facilities. Abbreviations: PBF, performance-based financing; IHVs, integrated household visit. Source: http://www.fbrburkina.org/data/element/16.

IHVs: Under PBF, providers were required to adopt new procedures and forms for conducting IHVs. Participants pointed to the increased number of IHVs conducted over time as one of the main positive effects of PBF (Figure 4). Providers across facilities often received high quality scores for IHVs. During the data collection period, however, we did not witness a single provider conduct a proper IHV, despite the fact that providers reported the maximum number of paid IHVs. Observation showed that providers used various strategies to manipulate the actual conduct of IHVs, which were considered “well paid.” Providers in at least 3 facilities falsified IHV forms and conducted IHVs of low quality. For example, providers filled out IHV forms minutes before PBF audits and falsified their content, including signatures of individuals purported to have been present, dates of appointments, and household analyses. The forms were sometimes filled out by individuals who were either not present during the reported visits nor even qualified to conduct them. In case 3, a midwife used women in the maternity ward to complete the forms rapidly without actually visiting their households. Another nurse conducted 8 household visits in 3 hours, despite reports that each visit takes one hour. The content of the lengthy IHVs forms was often superficial, citing the same strengths, weaknesses, causes, and plans across all households. PBF managers confirmed that they observed “fraud” and “major abuses” regarding IHVs and attempted various strategies to resolve this issue (eg, putting a cap on the number paid, suspending the purchase of IHVs).

**Figure 5:**
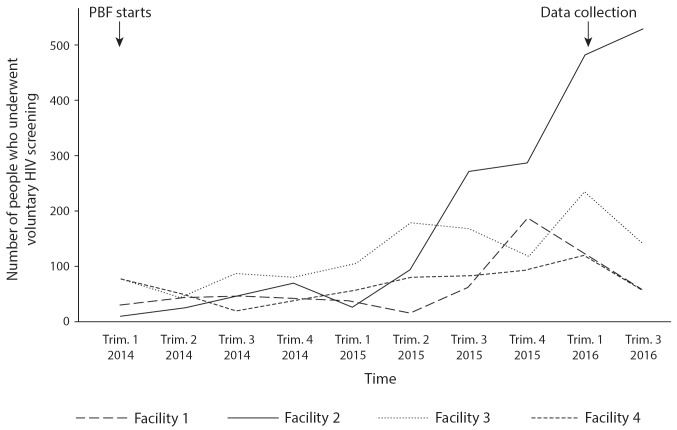
Number of People Who Underwent Voluntary HIV Screening Over Time. Abbreviation: PBF, performance-based financing. Source: http://www.fbrburkina.org/data/element/17.

HIV screening: Quantitative data suggests an increase in the number of voluntary screenings in 2015 and early 2016 (Figure 5). However, qualitative data suggested that providers falsely reported HIV screenings. Observation revealed few HIV screenings during consultations across facilities. In case 2, the facility with the highest number of screenings, observation revealed that providers prepared for a PBF audit by creating a new HIV register to report voluntary screenings for past patients and ensuring dates were concordant to avoid looking suspicious. PBF managers explained that “over declarations” were indicated by non-concordance between the stock of reagents available in the country and the number of reported screenings. To discourage abuses, the unit fee for this activity was lowered. PBF auditors became stricter, verifying concordance between the quantity of reagents used and the number of people reported as screened. This partly explains the reduction in reported screenings seen in Figure 5 during the third trimester of 2016.

 Note: The data collection indicated in the graph refers to the present study.

 Consultations: False consultations were added for patients followed in the maternity ward in at least 2 facilities. For example, when preparing for a PBF audit, providers in case 2 realized they had conducted few consultations for healthy children between 12-23 months, so they simply added consultations for former patients. Similarly, in case 3, providers regularly added false prenatal consultations for: (1) pregnant women who missed their appointments; (2) postnatal consultations for women who gave birth in the facility; and (3) children treated for malnutrition. Expected consultation dates were filled out with a pen in advance and patient information was filled in even if they did not come.

“*The midwife was sitting on the bench with the registers for moderately and severely acute malnourished children. She recorded data for about ten additional malnourished children who weren’t there.... She had no health booklets in her hands and no children around her”* (Facility 3, observation notes).

####  Complacency, Collusion, and Complicity

 There was complacency, collusion, and complicity between providers and managers around manipulating data to improve PBF scores. In all facilities, providers regularly witnessed each other falsifying registers without intervening. Supervisors were sometimes directly or indirectly involved in data falsification and manipulation. In facility B, before a quality audit, a district-level manager asked providers to place a trash container in front of the facility and to wear their lab coats. One PBF auditor also told the medication manager how to prepare receipts that would meet PBF criteria without giving these receipts to patients who purchased medication. In facility 1, a district supervisor advised providers to report a single absence so they could meet PBF criteria without having to report real absences. In facility 3, the head nurse encouraged the midwife to treat children as severely malnourished (an incentivized service), regardless of her clinical assessment. In all these examples, participants explicitly referred to PBF to justify their behaviour.

 Under PBF, the different healthcare system levels received performance-based payments. The performance of one level (eg, facilities) influenced that of others (eg, districts). Thus, managers, some of whom were PBF auditors, had vested interests in protecting facilities. In 28 months of implementation, no district management team reported any fraud in PBF facilities. No sanctions were given for data falsification in any intervention district. This is consistent with the broader social system, wherein providers have a history of mutual protection.

“*The supervisor said, It’s not possible that no one was absent during the month! Chief [nurse], you have to take the hit yourself and put yourself down for one day absent, just one day, so we [district management team] can get our 65 points! We got zero in the last trimester because of that. It sent shivers down our spine!”* (Facility 1, observation notes).

###  Unanticipated and Undesirable

####  Teaching Trainees Improper Practices

 Trainees doing internships in 4 PBF facilities often witnessed or participated in the falsification of registers to increase PBF scores. In at least 2 of these facilities, providers showed trainees tricks to avoid detection, such as filling out partographs in reverse, and ensuring consistency in information, and even handwriting, across registers.

“*The midwife arbitrarily changed the register filled out by the trainee in an earlier consultation, telling her, ‘Everything must be filled. Everything! Otherwise, it’s zero! They don’t care about you. PBF makes us write a lot. Too much! All information needs to be consistent. Otherwise, they know you want to cheat, but that you can’t’*” (Facility 4, observation notes).

####  Overwhelming Paperwork

 PBF considerably increased the paperwork load. While many registers existed prior to the PBF implementation, they were generally neglected in day-to-day practice. With PBF, some registers were modified to collect more information (eg, providers’ signatures and qualifications). Other documents were added for PBF purposes (eg, performance improvement plans, index tools, household visit forms). Consequently, the majority of providers across facilities complained that PBF required them to write “too much,” considering the staff shortages. To illustrate this, one head nurse exclaimed, “PBF is ink!”

####  Pursuit of Narrow Performance Indicators

 PBF auditors and managers sometimes focused on narrow/specific performance indicators that were perceived locally as irrelevant in the context, unrealistic, or too costly. For example, one PBF indicator referred to having a fence around the facility. No facilities were fenced, so auditors repeatedly recommended fence-building. At a national meeting, the district management team even presented the lack of fences as the primary difficulty relating to quality of care, exhibiting a “tunnel vision” focused on phenomena that were quantifiable in the performance measurement scheme. Under pressure, providers often included “building a fence” or “documenting facility boundaries” as objectives in performance improvement plans. However, most providers interviewed explicitly expressed low buy-in or disagreement with these objectives.

####  Manipulation of Index Tools

 With PBF, facilities were required to fill out a financial planning instrument, the index tool, each month to determine the premiums each worker should receive. The amount available for staff premiums depended on the characteristics of the facility (eg, revenues, expenses, savings) and the healthcare staff (eg, qualifications, years of experience, absences, individual evaluation scores). In most facilities, head nurses and staff manipulated data in the index tool for their own financial gain by either: (1) reducing the number of years of experience of other staff members (sometimes illiterate); (2) reporting the wrong staff qualification category of other staff members; (3) lowering a staff member’s individual evaluation score; (4) artificially inflating planned expenses to keep money for themselves; (5) under-reporting real expenditures to have more funds available for staff premiums; and (6) failing to report actual absences.

“*PBF is tactical. If we buy another [childbirth] delivery table, there will be nothing left for the workers”* (Facility 3, provider, observation notes).

“*They [providers] deliberately decided not to buy drugs to increase their profit margin and thereby increase their premium”* (National manager, discussion).

####  Tensions and Conflicts Related to Index Tools

 The index tool caused tensions and conflicts among stakeholders. First, providers were frustrated to learn they were receiving a considerably lower percentage of revenues as premiums than in the pre-pilot study. At the time of data collection, the index tools explicitly stipulated that the percentage of premiums given to providers should not exceed 30% of a facility’ revenues. Second, some providers were dissatisfied with the points attributed for different levels of responsibility. The tool automatically gave head nurses 20 points, which did not necessarily reflect the workload distribution. Third, the lack of transparency of some head nurses when filling out the index tool often provoked internal conflicts. Lastly, in most healthcare facilities, participants reported that the lack of formal inclusion of community representatives in index tools caused dissatisfaction, conflicts, and even demotivation. In PBF, community representatives involved in the management committee were required to update documentation, purchase medication, maintain the outside premises, withdraw subsidies from banks, etc. Participants revealed how this devalued their work, stating that they were “excluded,” “not part of the team,” “not important among these people,” and “doing nothing to increase subsidies.”

####  Frustrations for Providers not Eligible for Quality Points

 With PBF, medical registers were modified so that providers specified their names and qualifications. Some services provided by certain categories of providers were not eligible for quality points. However, this criterion clashed with local practices. For example, prior to PBF, itinerant health workers (trained to conduct health promotion activities), and birth attendants (trained to provide support in maternity wards) routinely treated patients by themselves. During quality audits, however, some of their consultations automatically received a score of zero under the justification that they were “unqualified.” Itinerant health workers and birth attendants expressed frustration at this perceived injustice. Most providers contested this evaluation criterion, arguing that it was not adapted to the local context, given: (1) staff shortages and the difficulty of hiring staff; (2) these workers’ life-saving work; (3) head nurses’ mobility; and (4) the fact that all providers followed the same diagnostic and treatment guide.

 In 5 facilities, providers developed strategies to systematically falsify the identities of unqualified providers who treated patients. Itinerant health workers, birth attendants, and trainees delivered services but left the signature/qualification columns blank. Later, qualified providers signed their names and qualifications despite their absence during these consultations.

*“What hurts me with PBF is that our actions are not considered quality. This morning, I did a delivery and it went very well, but it’s not considered quality. Yet I do the same acts. So, the midwife or head nurse will sign the register. It’s not fair” *(Facility 2, itinerary health agent, observation notes).

####  Staff’s Dissatisfaction and Demotivation Due to Payment Delays

 While most participants indicated that PBF was more advantageous than previous practices, the long payment delays were a source of dissatisfaction and demotivation across all facilities. During the study period, payment delays for quantity-related subsidies were over 6 months, while those for quality-related bonuses were over 16 months. The quality improvement bonus was cancelled altogether. Many participants reported that delays were getting longer and PBF was losing its dynamism. Their dissatisfaction was exacerbated by the lack of communication regarding the causes of delays and their increased workload.

####  Suboptimal Planning Due to Payment Delays

 Every month, providers were required to plan expenditures using PBF management tools (eg, performance improvement plan, index tool). Providers were required to fill out the index tool as if monthly subsidies were already available. However, long payment delays limited the practical application of PBF management tools across all the facilities. Numerous participants described this as having “virtual money” that could not actually be spent.

“*The problem with the performance improvement plan is that you plan activities but then don’t have the means to do them because the transfers are late” *(Facility 3, provider, interview 72).

####  Financial Issues

 Overall, the facilities’ management teams reported having more funds than before PBF. These were used, for example, to replenish medication stocks. However, many participants complained that payment delays caused financial issues over time, especially for small or vulnerable facilities. Under the PBF principle of managerial autonomy, many expenses previously covered by district management teams had been transferred to facilities, such as photocopying, meetings, training costs, mattresses, and carbonized receipts. PBF also generated specific expenses, such as food and drinks for auditors, copies of longer forms and new documentation, PBF meetings, and materials recommended by auditors. While payment delays were a challenge for some facilities, the financial gaps were covered once subsidies were transferred.

####  Tensions Between Managerial Autonomy and Top-Down Control

 PBF increased facilities’ managerial autonomy by allowing providers to make expenditures up to 50 000 F CFA without the district managers’ approval and to recruit additional staff. At the same time, however, PBF was perceived as a directed, top-down approach. A few participants perceived PBF as a form of control. To prevent mismanagement, providers had to follow strict guidelines on how to spend revenues. Ninety percent of revenues from the drug depot had to be used to buy more medication. Providers could only take premiums if the facility’s savings covered operating costs for 90 days. In theory, premiums for workers could not exceed 30% of revenues, but in reality, PBF auditors and district management teams constantly reduced this percentage in the index tool (eg, to 12%–17%) to increase bank reserves. They often modified the index tool content without consulting providers or obtaining consent. Moreover, coercive measures were used to force the adoption of PBF. Facilities that did not follow PBF guidelines or whose performance was not adequate were threatened with suspension. Also, head nurses were required to comply with PBF guidelines as part of their mandate. Thus, there was growing tension between the principle of managerial autonomy and control.

“*The monthly validation of the index tool is somewhat contrary to the principles of autonomy, but they were forced to go there because there were abuses” *(Facility 3, provider, interview 72).

####  Activities Delayed and Reduced Due to Gradual Withdrawal of Other Funding

PBF entailed a reduction in other sources of funding from the national level. Certain activities were now required to be covered by PBF subsidies and bonuses. The *Programme d’appui au développement sanitaire* (PADS – program to support health development) managed a common basket that combined funds from the government and financial partners to support district management teams. Facing financial difficulties, the PADS stopped allocating certain funds to PBF districts, reallocating them to non-PBF districts. Participants in multiple facilities perceived this as reduction of the state’s commitment.

 Many providers believed the changes in funding modalities caused delays and reductions in the number and duration of activities, including meetings and training sessions. According to the PBF principle of managerial autonomy, district management teams and head nurses were expected to assess providers’ needs and use subsidies to organize activities, but this did not happen as planned. Many activities previously funded through the PADS were either not organized in a timely fashion or were shortened, possibly affecting their quality. This upset providers, who previously had received per diems when attending these activities.

####  A “Budgetivorous” Intervention

 Participants in healthcare facilities and at the national level expressed concerns about high costs of PBF related to audits, meetings, registers, etc. Some described the intervention as “budgetivorous,” arguing that it disproportionately consumed budgets. Many questioned its financial sustainability.

“*PBF is expensive! … compared to non-PBF districts, budgets range from equal to 5 or 6 times higher. The results are not proportional. So, we may have to look at how PBF should be adapted to the State budget” *(National manager, interview 105).

“*PBF eats up budgets. It’s making us spend too much” *(Facility 1, provider, interview 19).

## Discussion

This theory-driven study makes a unique contribution to the literature by documenting a neglected topic, the unintended consequences of PBF in an LMIC. The vast amount of data analyzed will give stakeholders a more comprehensive picture of PBF consequences in a real-life setting. Consistent with Rogers’ diffusion of innovations theory, the results showed that PBF led to both desirable and undesirable unintended consequences, with the latter largely outweighing the former. This was partly due to the fact that some desirable consequences were considered to have been intended by program planners and were therefore outside the focus of this study. For example, we found some evidence that PBF was related to (1) feedback loops between supervisions and PBF audits, (2) some improvement in providers’ knowledge, (3) increased social pressure for performance improvement, and (4) improvement of staff’s socioeconomic well-being. While these were not addressed in the implementation guides, PBF experts considered them to be intended according to the “spirit of the intervention” or its ideas.^21^ This highlights the importance of going beyond implementation guides to decipher between intended and unintended consequences.

Moreover, the classification in Table 3 showed that almost all unintended consequences were primarily related to processes (ie, intervention roll-out) rather than outcomes. This may be, to some extent, because the intervention model identified providers as the locus of behavioural changes,^44^ providers implemented few creative strategies that affected outputs, and communities were not well informed about or involved in PBF.

The results are consistent with the diffusion of innovations theory, which stipulates that while financial incentives may accelerate an innovation’s adoption, the quality of adoption decisions may be low.^19^ In this study, providers were incentivized to report increases in care, but many services were not actually delivered as reported, limiting PBF’s potential impact. Furthermore, some providers were fixated on performance measures and subsidies rather than on underlying objectives, again suggesting they did not always internalize the rationale linked to improving certain dimensions of services (eg, patient confidentiality). These results suggest that, given providers’ discretionary power in carrying out interventions, healthcare managers may have to find strategies to improve local actors’ adherence to the underlying objectives of PBF to truly improve care.^45^

Findings from this study raise important methodological considerations for the overall work of assessing PBF impact. While reported quantitative performance data suggested healthcare services had improved considerably, observations revealed that registers were often falsified to artificially enhance performance. The contrast between qualitative and quantitative data shown in this study highlights the risk of relying solely on one method to understand the effects of complex interventions such as PBF. The interpretation of quantitative performance data is more meaningful when implementation processes and local adaptations are considered. In Burkina Faso, the World Bank had not initially planned to conduct a process evaluation and only decided to do so after the impact evaluation. Like Cataldo and Kielmann,^42^ we believe PBF researchers should place more emphasis on spending time in the field, gaining trust, building rapport, conducting observation, and sustaining dialogue with participants to reap rich data that can further the understanding of how stakeholders respond to PBF and its impacts. Such approaches are crucial to send to the right policy signals to decision-makers.

An important question is whether PBF is responsible for the falsification of registers. Prior evidence indicates some falsification occurred in the absence of PBF.^46^ During data collection, we did witness some falsification unrelated to PBF. Following the diffusion of innovations theory,^19^ we considered such behaviours to be part of past experiences and local practices that influence how local adopters re-invent innovations such as PBF. Nevertheless, the rich data produced through our long-term involvement clearly suggested the existence of a link between PBF and the falsification of registers.^47^ We were able to capture the link between PBF and falsification (as well as other unintended consequences) based on an in-depth understanding of meanings, contexts, and processes.^48^

This study builds on our previous work on the unintended consequences of community verifications for PBF in Burkina Faso.^14^ Integrating both articles highlights that the anticipation of community verifications was not sufficient to dissuade providers from falsifying registers. Together, the articles also reveal weaknesses in the overall verification system. Providers were routinely falsifying data to increase performance scores, but community verifications were not able to clearly detect this falsification due to the numerous implementation challenges during the community verifications (eg, difficulty retracing patients, falsification of community verifiers in charge of tracing patients). In 28 months, no sanctions were given for the falsification of registers. This is similar to what has been observed in Niger where impunity prevails for professional misconduct and the only “sanction” applied in practice is to move a provider to another site.^49^

Many unintended consequences detected in our work resonate with studies conducted elsewhere. A study in Rwanda also reported a fixation on performance measures.^10^ Participants argued that when an incentive is offered for a precise indicator, it becomes “dissociated” from its very meaning and loses its rationale. That study also noted paperwork overload. Participants explained that time limitations forced them to choose between essential activities and those required for rewards (eg, paperwork). Consistent with our findings, performance indicators were often falsified to improve reported results.^10^ High concordance between providers’ declared numbers and PBF auditors’ validated numbers, as studied by Kuunibe and colleagues,^50^ does not rule out falsification. Our results showed deliberate falsification often occurred upstream, directly in the medical registers, and could not easily be detected by PBF auditors, thereby raising questions regarding the effectiveness of audits.

Moreover, many undesirable consequences regarding the payment and distribution of subsidies are consistent with existing evidence. First, research suggests workers lose motivation when incentive agreements are not respected.^51,52^ Such implementation lapses go against the intervention theory, which relies on financial incentives to motivate staff. Second, regarding the demotivation of community representatives who did not receive premiums, participants in a study in Tanzania warned against solely rewarding providers, as they often have to collaborate with community leaders.^9^ Third, studies in Benin, Rwanda, and Burkina Faso showed that providers were concerned about “unfair” distribution of rewards.^10,53,54^ As in our study, it was not always those producing the greatest results who obtained the highest compensation. Lastly, our study echoed findings in Benin, where providers suspected their hierarchic superiors of monopolizing premiums.^54^ In Burkina Faso, even Ministry of Health senior executives requested and obtained PBF premiums.

This study has important implications for global health organizations and policy-makers in LMICs. In coming years, many LMIC governments will pursue PBF through new funding agreements with the Global Financing Facility and the World Bank. This is already underway in Burkina Faso.^55^ Given their scope and breadth, we advise careful consideration of the undesirable consequences of PBF before pursuing or scaling up the intervention.

 When discussing preliminary results with high-level stakeholders in Burkina Faso, some revealed that they were already aware of many unintended consequences reported in this study. Deliberations were already underway to resolve some of them. For example, they planned to stop purchasing IHVs due to the falsification. They also planned to start paying community health worker premiums to increase their satisfaction levels. However, the later was not materialized due to costs. This suggests that some unintended consequences may be addressed while others may have to be accepted as trade-offs if the intervention is to be pursued. Actions can be decided on a case by case basis. It should be noted, however, that local stakeholders in Burkina Faso reported having little room for manoeuvre to adapt this World-Bank intervention.

The study has implications for future research. We hope the framework and methods will stimulate research on unintended consequences of PBF in other settings and of other complex health interventions in LMICs to produce more comprehensive evidence to improve population health. As PBF expands, future research could also examine whether it triggers unintended consequences in other sectors, such as education, in LMICs.^56^

###  Limitations

 We recognize the potential limitations of the study. First, the 6 facilities were in only 2 districts, which limits the transferability of findings. Although prolonged observation limited the number of facilities we could include, it produced rich findings with high internal validity. Moreover, member checks with stakeholders at the national level confirmed many of the results. Second, we encountered a language barrier due to the large number of languages spoken in Burkina Faso. We used local interpreters to conduct 15 interviews. The researchers’ background helped minimize the language barrier. The first author’s mother tongue is French, a language regularly spoken between providers. She also took courses to learn Dioula and participated in a 4-month immersion program in a Dioula-speaking area. The second author also spoke French and learned Dioula while living in Burkina Faso. The last author also has a good understanding of the context with over 20 years of experience in the region. A third limitation is that the quantitative data were used for descriptive statistics only. We did not perform statistical tests, which limits the depth of these complementary analyses.

## Conclusion

 PBF is widely implemented in many LMICs to improve healthcare system performance. This multiple case study provided new insights into its unintended consequences and their contributing factors. Results showed PBF led to important unintended consequences in primary healthcare facilities. Most unintended consequences were undesirable and could jeopardize the intervention. With this evidence, policy-makers may be able to develop strategies to avoid or minimize unintended consequences. Others may be accepted as trade-offs. More evidence is needed on unintended consequences of complex health interventions to help achieve universal health coverage.

## Acknowledgements

The authors are grateful to Dr. Manuela De Allegri for her valuable input to this manuscript. The authors also express their gratitude to the members of the PBF technical service, including Dr. Philippe Compaoré and Dr. Aloys Zongo, the district-level managers, and all participants for their generous contributions to this study. This study benefited from the data on the Ministry of Health’s PBF portal, so we thank all actors involved in collecting and reporting this data (http://www.fbrburkina.org/). The authors would also like to thank Donna Riley for her editing support on a previous version of the manuscript and Guillaume Edger for help with the graphic design.

## Ethical issues

 The protocol was approved by the ethics committees in Burkina Faso (deliberation N_ 2015-12-07) and at the University of Montreal Hospital Research Centre (CE 13.358).

## Competing interests

 VR was a co-researcher on the baseline study of the impact evaluation of PBF in Burkina Faso. However, he received no salary from the funder (World Bank) for this activity. The authors have no conflicts of interest regarding the publication of this paper.

## Authors’ contributions

 AMTT conceived the study protocol, collected and analyzed the data, and wrote the first draft of the manuscript. VR helped conceive the study protocol, interpret the results, and critically revised the manuscript. IAGG collected part of the data, contributed to the analysis, and reviewed the manuscript. All authors read and approved the final manuscript.

## Authors’ affiliations

^1^École de santé publique de l’Université de Montréal, Montreal, QC, Canada. ^2^Association Action Gouvernance Intégration Renforcement (AGIR), Ouagadougou, Burkina Faso. ^3^IRD (French Institute for Research on Sustainable Development), CEPED, Université de Paris, Paris, France.

## Finding

 This work was supported by a research award funded by the International Development Research Centre (Project No. 107759-99906075-009). AMTT received a training bursary from the Canadian Institutes of Health Research (CIHR). The research project is part of the “Community research studies and interventions for health equity in Burkina Faso” (ROH-115213), supported by CIHR. The authors also received funding from the University of Montreal Public Health Research Institute. Sponsors did not have a role in the study design; the collection, analysis, and interpretation of data; the writing of the report; and the decision to submit the article for publication.

## Supplementary files


Supplementary file 1. Examples of Unit Prices for PBF Indicators for Quantity Verifications.

Supplementary file 2. Dimensions of Technical Quality of Care Assessed Every Trimester.

Supplementary file 3. Examples of Semi-structured Interview Questions.

Supplementary file 4. Justification of the Classification of Consequences as Anticipated Versus Unanticipated According to Intervention Guides.

Supplementary file 5. Additional Examples of the Falsification of Medical Registers and Documentation.
