# Supplementary material for: An Exploration of the Unintended Consequences of Performance-Based Financing in 6 Primary Healthcare Facilities in Burkina Faso
Source: Int J Health Policy Manag. 2020 Jun 23;11(2):145–59. doi: 10.34172/ijhpm.2020.83 (PMC9278611; doi:10.34172/ijhpm.2020.83)
Supplement: Supplementary file 1 — Examples of Unit Prices for PBF Indicators for Quantity Verifications. [file ijhpm-11-145-s001.pdf]

Supplementary file 1. Examples of Unit Prices for PBF Indicators for Quantity Verifications

		**Unit prices in CFA francs**			
	**Indicators**	**Facility 1**	**Facility 2**	**Facility 3**	**Facility 4**
1	Number of new patients aged 5 years and older seen in curative consultation	140	120	140	130
2	Number of new patients aged less than 5 years seen in curative consultation	210	180	210	200
3	Number of days of patients place under observation	350	300	350	330
4	Number of counter-references obtained from hospitals accepting referred patients	1 400	1 200	1 400	1 300
5	Number of children completely vaccinated	430	360	320	390
6	Number of pregnant women who received tetanus toxoid at least twice during the month	350	300	350	330
7	Number of prenatal consultations conducted in the facility during the month	560	480	560	520
8	Number of postnatal consultations conducted in the facility during the month (D6-D8 and W6-W8)	700	600	700	650
9	Number of deliveries conducted with partograph during the month	2 100	1 800	2 100	1 950
10	Number of women (old and new) seen during the month in family planning consultation and using long-term contraceptive methods (DUI and implant)	700	600	700	650
11	Number of women (old and new) seen during the month in family planning consultation and using oral contraceptives or injectables	1 400	1 200	1 400	1 300
12	Number of newly enrolled patients 0-11 months seen in healthy child consultations	140	120	140	130
13	Number of children aged 12-23 months seen in healthy child consultations	350	300	350	330
14	Number of children aged 6-59 months treated for moderate acute malnutrition	420	360	420	390
15	Number of children aged 6-59 months treated for severe acute malnutrition	1 050	900	1 050	980
16	Number of integrated household visits conducted	4 200	3 600	4 200	3 900
17	Number of people who underwent voluntary screening for HIV (aside from those screened in the context of mother-to-child transmission)	700	600	700	650

18	Number of pregnant women and partners who underwent HIV screening in the context of mother-to-child-transmission	700	600	700	650
19	Number of HIV+ mothers treated with antiretrovirals	3 500	3 000	3 500	3250
20	Number of children born from HIV+ mothers followed	4 200	3 600	4 200	3 900
21	Number of people living with HIV/AIDS treated with antiretrovirals	1 400	1 200	1 400	1 300
22	Number of cases of smear-positive pulmonary tuberculosis (new cases or relapses)	8 400	7 200	8 400	7 800
23	Number of tuberculosis cases (all forms) treated and declared cured or treatment ended	11 900	10 200	11 900	11 050
Note: 1 USD = 581.18 CFA francs					

Abbreviation: PBF, performance-based financing.
