# Supplementary material for: An Exploration of the Unintended Consequences of Performance-Based Financing in 6 Primary Healthcare Facilities in Burkina Faso
Source: Int J Health Policy Manag. 2020 Jun 23;11(2):145–59. doi: 10.34172/ijhpm.2020.83 (PMC9278611; doi:10.34172/ijhpm.2020.83)
Supplement: Supplementary file 2 — Dimensions of Technical Quality of Care Assessed Every Trimester. [file ijhpm-11-145-s002.pdf]

Supplementary file 2. Dimensions of Technical Quality of Care Assessed Every Trimester

	**Verified area or activity**	**Maximum score possible (case 1)**
1	General indicators	70
2	Conditions of reception/waiting area for patients	50
3	Availability of medical technical equipment	110
4	Bookkeeping	60
5	Management of medications, consumables, and inputs	150
6	Respect of norms related to stocking and storing consumables and to conducting certain activities	140
7	Financial management	80
8	Prevention of infections (hygiene and sanitation)	120
9	Performance improvement plan	60
10	Household visits	80
11	Care for new patients aged 5 years and older seen in curative consultations	100
12	Care for sick children aged 2 months to less than 5 years	250
13	Patients under observation	100
14	Vaccination of children aged 0-11 months	50
15	Prenatal consultations	160
16	Postnatal consultations	90
17	Births	250
18	Family planning	80
19	Consultations for healthy children aged 0-11 months	55
20	Consultations for healthy children aged 12-23 months	50
21	Care for children aged 6-59 months with moderate acute malnutrition	80
22	Care for children aged 6-59 months with severe acute malnutrition without complications	115
23	Screening for HIV infection	60
24	Care for HIV-positive pregnant women	25
25	Application of the PMTCT protocol for newborns of HIV positive mothers	25
26	Monitoring for people living with HIV under ARV	25
27	Screening for tuberculosis	50
28	Care for treated and cured cases of TB	100
	**Total**	**2 565**
