# Supplementary material for: An Exploration of the Unintended Consequences of Performance-Based Financing in 6 Primary Healthcare Facilities in Burkina Faso
Source: Int J Health Policy Manag. 2020 Jun 23;11(2):145–59. doi: 10.34172/ijhpm.2020.83 (PMC9278611; doi:10.34172/ijhpm.2020.83)
Supplement: Supplementary file 3 — Examples of Semi-structured Interview Questions. [file ijhpm-11-145-s003.pdf]

Supplementary file 3. Examples of Semi-structured Interview Questions

Characteristics and perception of actors

1. What is your current position?
2. How long have you been in this role?
3. What is your involvement in PBF?
4. Could you describe how PBF activities are carried out in your organization?
5. What do you think of PBF (eg, advantages and disadvantages)?
    - nature of incentives
    - performance indicators
    - verification process
    - incentive distribution
6. How did you first hear about the PBF? What was your reaction when you first heard about it?
7. Who was involved in developing or adapting the intervention?
8. Does everyone support PBF? What reactions did you observe?

Nature of the social system

9. Is PBF adapted to the local context?
    - health system (eg, organizational capacity, resources)
    - local needs
    - local beliefs and values
10. What are the similarities and differences between you and the promoters of PBF?
11. Do your previous experiences or relationships with donors or promoters of PBF influence your perception of the intervention?
12. What factors influence the implementation process of the intervention?
13. How is information about PBF communicated between the different actors involved?
14. Do you receive any comments or advice about your performance after quality and quantity verifications?

Nature and use of innovation

15. Before the implementation of PBF, what strategies were used to motivate health workers to improve care? Is the new system more useful than the old system?
16. Is PBF easy to understand and implement? What are the difficulties?
17. Did you have the opportunity to test the intervention and make suggestions for improvement?
18. Did the intervention solve some problems and meet your needs?
19. How well is the intervention adapted to what health workers are used to and expect?
20. Is the intervention flexible?
21. Have you changed the way that FBR activities are conducted?
22. How does the distribution of subsidies and bonuses work? What have you received so far?
23. Who benefits from the intervention? Who does not benefit?

Changes

24. What changes has the intervention caused?
    - organization of the health system
    - practices, behaviours or activities
    - governance or supervision
    - level of autonomy of health centres
    - working conditions (wages, number of hours worked) or staff absenteeism
    - quantity of healthcare (discuss possible increases, reductions or stagnation depending on the type of care)
    - quality of healthcare (discuss possible increases, reductions or stagnation depending on the type of care)
    - community outreach activities (eg, home visits)
    - relationships between actors (eg, conflict, collaboration)
    - power or social positions of actors
    - cultures, beliefs or values of actors
    - motivation
    - initiatives or strategies to increase the quality and quantity of care
    - care for vulnerable groups

- the revenues and expenses of health centres
- availability of or access to medical equipment
- the availability of or access to medicines
- migration or rotation of health staff
- communication between the different actors of the health system
- the participation of community members in the health system (eg, members of the management committee)
- population health
- laws
- technology
- unintended consequences (desirable or undesirable)

Temporality

25. How have the consequences of PBF changed over time?
26. How could PBF be improved in the future?
27. How do you think that PBF will have evolved in 10 years?
