# Supplementary material for: An Exploration of the Unintended Consequences of Performance-Based Financing in 6 Primary Healthcare Facilities in Burkina Faso
Source: Int J Health Policy Manag. 2020 Jun 23;11(2):145–59. doi: 10.34172/ijhpm.2020.83 (PMC9278611; doi:10.34172/ijhpm.2020.83)
Supplement: Supplementary file 4 — Justification of the Classification of Consequences as Anticipated Versus Unanticipated According to Intervention Guides. [file ijhpm-11-145-s004.pdf]

Supplementary file 4. Justification of the Classification of Consequences as Anticipated Versus Unanticipated According to Intervention Guides

Anticipated		Unanticipated
**Direct (Process)**	**Indirect (Outcome)**	**Direct (Process)**
		Limits on medication sales without consultations<br>➢ Not addressed in guides
Gaming<br>➢ "To strengthen the credibility of the auditing (quantitative and qualitative), a counter-audit is carried out at 6-month intervals by an external entity recruited for this purpose. It consists in verifying, in a sample of health facilities, that the audit was properly conducted."[30]<br>➢ "The development of the spirit of initiative. Actors are incited to put in place innovative strategies to address the challenges they face in terms of barriers to access and to the use of services.[40]<br>Fixation on indicators and subsidies<br>➢ "Derelictions occur when service providers neglect services that are not supported by incentives"[30]<br>Falsification of medical registers and documents<br>➢ "The results of the survey on the veracity of services may, in the event that fraud is detected, result in sanctions for the offending health facilities"[30]<br>➢ "Fraud refers to the observation, in the supporting documentation for an activity (curative, preventive, or promotional), of a different handwriting, a pen of a different tone or colour, excessive deletions, rampant use of "white-out," the creation of fictitious users, the reporting of acts or services that the user did not actually receive, etc."[30]<br>Complacency, collusion and complicity<br>➢ "To strengthen the credibility of the auditing (quantitative and qualitative), a counter-audit is carried out at 6-month intervals by an external entity recruited for this purpose. It consists in verifying, in a sample of health facilities, that the audit was properly conducted."[30]		Teaching trainees improper practices<br>➢ Not addressed in guides<br>Overwhelming paperwork<br>➢ Not addressed in guides<br>Pursuit of narrow performance indicators<br>➢ Not addressed in guides<br>Manipulation of the index tools<br>➢ Not addressed in guides. An optimistic view of the index tool is presented.<br>Tensions and conflicts related to index tools<br>➢ Not addressed in guides. An optimistic view of the index tool is presented.<br>Staff's dissatisfaction and demotivation due to payment delays<br>➢ Not addressed in guides. PBF is intended to improve motivation.<br>Suboptimal planning due to payment delays<br>➢ Not addressed in guides<br>Financial issues<br>➢ Not addressed in guides<br>Frustrations for providers not eligible for quality points<br>➢ Not addressed in guides<br>Tensions between managerial autonomy and trop-down control<br>➢ Not addressed in guides. PBF is intended to improve autonomy.<br>Activities delayed and reduced due to gradual withdrawal of other funding<br>➢ Not addressed in guides<br>A "budgetivorous" intervention<br>➢ Not addressed in guides
