# Supplementary material for: An Exploration of the Unintended Consequences of Performance-Based Financing in 6 Primary Healthcare Facilities in Burkina Faso
Source: Int J Health Policy Manag. 2020 Jun 23;11(2):145–59. doi: 10.34172/ijhpm.2020.83 (PMC9278611; doi:10.34172/ijhpm.2020.83)
Supplement: Supplementary file 5 — Additional Examples of the Falsification of Medical Registers and Documentation. [file ijhpm-11-145-s005.pdf]

Supplementary file 5. Additional Examples of the Falsification of Medical Registers and Documentation

Identity and qualification of providers: PBF was aimed at improving quality of care by incentivizing the provision of services by certain types of qualified providers. Managers modified registers so that providers could indicate their names and qualification for each service provided. Services provided by certain types of providers (eg, itinerant health workers) were not eligible for quality points, although they counted for quantity audits. This evaluation criterion clashed with the healthcare system context and local practices. Participants explained that nurses could not consult with all patients due to the quantity of work and the shortage of human resources. Thus, different types of providers were already in the habit of providing care beyond their official level of expertise to avoid leaving patients unattended.

In all but one facility, providers developed a range of strategies to falsify the identity and qualification of providers delivering services. Often, providers who did not qualify for quality points delivered the services as usual but left the signature and qualification columns blank in registers. Later, qualified providers (eg, nurses for curative consultations or midwives for maternal care) signed their names and qualifications, despite not having been present for those consultations. In case 2, the itinerant health worker, who consulted alone when the head nurse was absent, wrote patient information on a sheet of paper or in an old register. Upon his return, the head nurse transcribed consultation information into the real register that was audited for PBF. The head nurse corrected the information as needed and signed as the sole provider. This ensured that the handwriting was consistent for both the medical information and signature to avoid detection by PBF auditors. In case 3, the midwife and birth attendant systematically co-signed each service delivered in the maternity ward to ensure they met the evaluation criteria regarding provider qualification. These signatures were added subsequently even though they were not both present during consultations.

“The head nurse retranscribed all the consultations and signed as if he had provided the care. But he was in another town… He makes the corrections as he goes along.” (case2_observation)

Figure A. Illustration of the falsification of providers’ identity and qualification

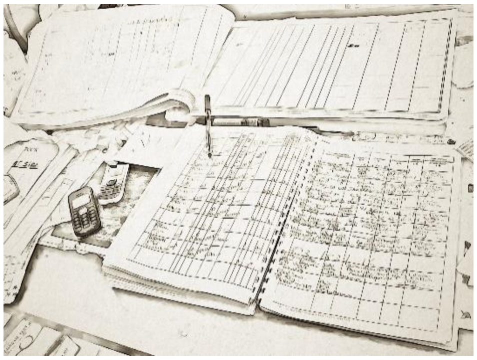

Description: Itinerant health workers used an old register (top) to record information on patient consultations. Later, the head nurse transcribed this medical information into the real register (bottom) and signed as the care provider to score quality points during PBF audits.

Dates: Consultations that did not respect the recommended appointment intervals received a score of zero in PBF audits. Consequently, providers across facilities sometimes falsified consultation dates. Participants explained that this rigid criterion was not adapted to the local context because illiterate patients often made mistakes on dates and the long distances made it difficult for them to return another day. Providers argued they should not be penalized for something over which they lacked control.

Prescriptions: Quality points were deducted if providers prescribed medications that did not comply with the diagnostics and treatments guide. Thus, the medication prescriptions reported in medical registers sometimes differed from the medication actually prescribed. This enabled providers to increase quality scores while prescribing what they wished.

“What we have found is that sometimes... in the register, [providers] prescribe what the Guide recommends, but in reality, they prescribe something else.” (National manager_106, interview)

Other health data: The falsification of medical data to increase PBF subsidies affected health statistics beyond the intervention. First, providers falsified the number of consultations directly in the medical registers, which are also used to collect data for the National Health Information System. Every month, providers used the falsified medical registers to fill out the facilities’ monthly reports, which were transferred to the districts’ Health Information and Epidemiological Surveillance Centres. District teams then entered the falsified statistics into their system, which is used to monitor population health and plan interventions.

Moreover, in case 3, providers who were not familiar with PBF evaluation criteria but who were under pressure to improve performance scores, falsified services that were not covered by PBF. For example, PBF only paid for newly enrolled healthy children seen in consultation but false consultations were also added for returning infants.
